# IRF4 p.T95R combined immunodeficiency: Clinical features and transplant outcomes

**DOI:** 10.70962/jhi.20260069

**Published:** 2026-10-09

**Authors:** Mattison P. Stojcic, Makenzie A. Manning, Miguel Galicchio, Manfred Hönig, Despina Moshous, Sven Kracker, Felipe Suarez, Ruben Martinez-Barricarte, Aidé Tamara Staines-Boone, Yuridia Salazar Gálvez, Edgar-Domingo Cordero-Chura, Estefany-Graciela Mamani-Velasquez, Jian Cui, Jeremie Rosain, Pandiarajan Vignesh, Sergio D. Rosenzweig, Klaus Schwarz, Catherine M. Biggs, Jacob Rozmus, Stuart E. Turvey

**Affiliations:** 1 Department of Pediatrics, BC Children’s Hospital, The University of British Columbia, Vancouver, Canada; 2 Experimental Medicine Program, Faculty of Medicine, The University of British Columbia, Vancouver, Canada; 3 Allergy and Immunology Service, Hospital de Niños VJ Vilela, Rosario, Argentina; 4 Department of Pediatrics and Adolescent Medicine, University Medical Center Ulm, Ulm, Germany; 5 Pediatric Immunology, Hematology and Rheumatology Department, Necker-Enfants Malades University Hospital, Assistance Publique Hôpitaux de Paris, Paris, France; 6 Laboratory “Genome Dynamics in Human Diseases,” INSERM UMR 1163, Imagine Institute, Université Paris Cité, Paris, France; 7 Laboratory of Lymphocyte Activation and Susceptibility to EBV Infection, INSERM UMR 1163, Imagine Institute, Université Paris Cité, Paris, France; 8 Department of Hématologie, Necker - Enfants Malades Hospital, Assistance Publique des Hopitaux de Paris (Institut IMAGINE INSERM U1163), Université Paris Cité, Paris, France; 9 Division of Genetic Medicine and Clinical Pharmacology, Department of Medicine, Vanderbilt Genetics Institute, Vanderbilt University Medical Center, Nashville, TN, USA; 10 Division of Molecular Pathogenesis, Department of Pathology, Microbiology, and Immunology, Vanderbilt Center for Immunobiology, Vanderbilt Institute for Infection, Immunology, and Inflammation, Vanderbilt University Medical Center, Nashville, TN, USA; 11 Immunology Service, Hospital de Especialidades Unidad Médica de Alta Especialidad, 25 del Instituto Mexicano del Seguro Social, Monterrey, México; 12 Pediatric Allergy and Immunology Department, Dr Ovidio Aliaga Uría Children’s Hospital, La Paz, Bolivia; 13 Study Center for Primary Immunodeficiencies, Necker Hospital for Sick Children, Assistance Publique Hôpitaux de Paris, Paris, France; 14 Pediatric Allergy Immunology Unit, Department of Pediatrics, Post Graduate Institute of Medical Education and Research, Chandigarh, India; 15 Immunology Service, Department of Laboratory Medicine, Clinical Center, National Institutes of Health, Bethesda, MD, USA; 16 Institute for Transfusion Medicine, University of Ulm, Ulm, Germany; 17 Institute for Clinical Transfusion Medicine and Immunogenetics Ulm, German Red Cross Blood Transfusion Service Baden-Württemberg-Hessen and University Hospital Ulm, Ulm, Germany; 18 Université Paris Cité, Institut Imagine, Laboratory of Human Genetics of Infectious Diseases, Necker Branch, INSERM UMR 1163, Paris, France.

## Abstract

IRF4 is a transcription factor within the interferon regulatory factor family that controls key aspects of lymphocyte differentiation and adaptive immunity. The heterozygous *IRF4* p.Thr95Arg (p.T95R) variant affecting the DNA-binding domain was recently identified to cause a new inborn error of immunity, but the longitudinal clinical course and therapeutic implications remain incompletely defined. We performed detailed clinical characterization and follow-up of all 10 known patients. Each presented with early-onset combined immunodeficiency characterized by hypo- or agammaglobulinemia and marked susceptibility to opportunistic infections. Pulmonary disease was universal, and gastrointestinal involvement was associated with a more severe clinical course. Four patients underwent hematopoietic stem cell transplantation; two survived, and two died. Notably, both deaths occurred in patients transplanted in the setting of active infection. These findings define IRF4 p.T95R as a profound combined immunodeficiency with high infectious burden and demonstrate that disease severity and intervention timing may contribute to transplant outcomes, supporting early diagnosis and timely transplantation before uncontrolled infection.

## Teaser

This study provides the first comprehensive description of the clinical phenotype and outcomes of all known patients with the heterozygous IRF4 p.T95R variant. Stojcic et al. analyze 10 affected individuals, identifying shared clinical features, treatment responses, and outcomes, and consider implications for hematopoietic stem cell transplantation.

## Graphical Abstract

**Figure:**
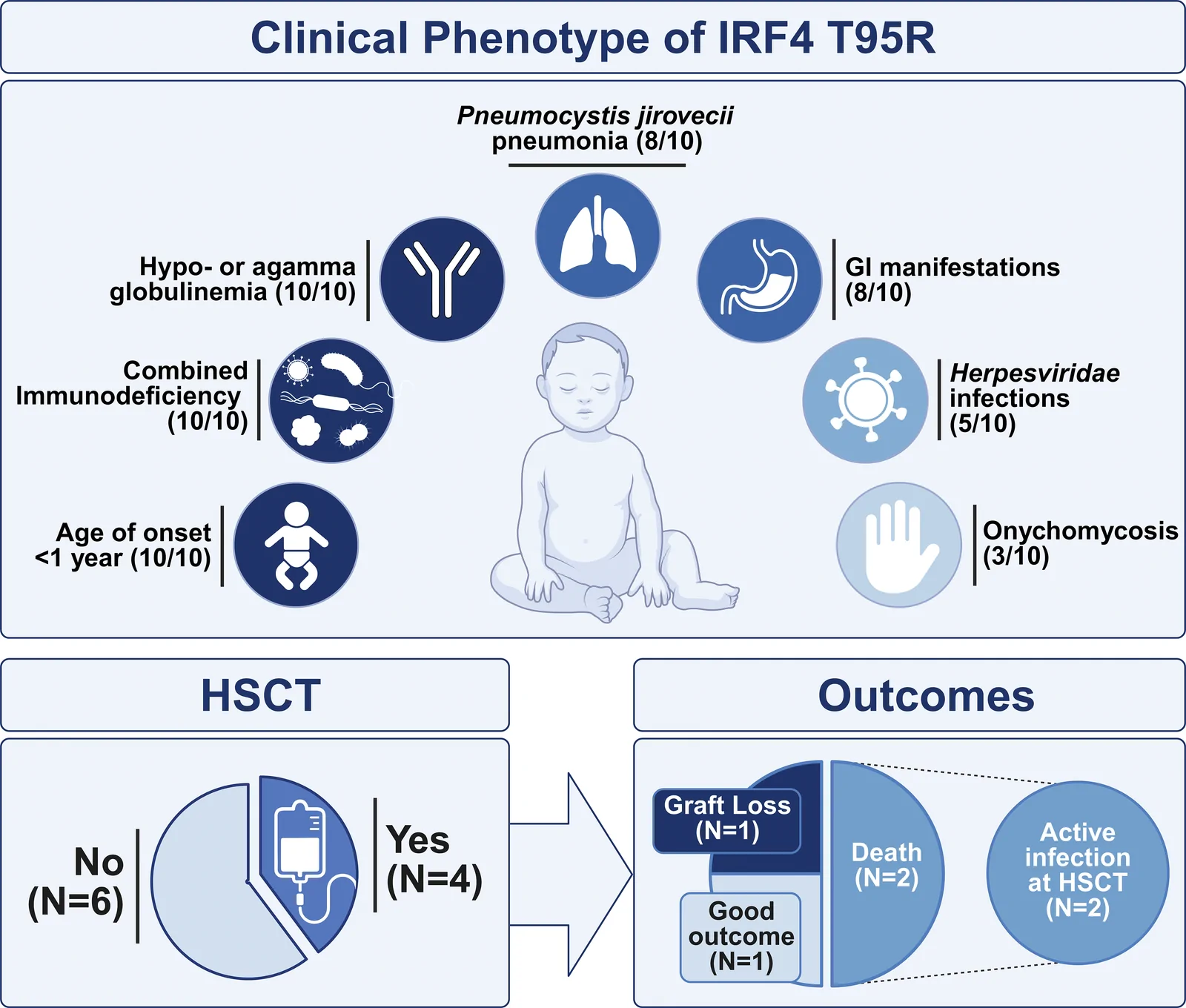

## Introduction

Human inborn errors of immunity (IEIs), which include primary immunodeficiencies, primary immune regulatory disorders, and primary atopic disorders, encompass a broad group of genetic disorders impairing immune function (1, 2, 3, 4). The phenotypes of the over 550 known IEIs span immunodeficiency, autoimmunity, autoinflammation, allergy, and malignancy (5). Tracking the clinical outcomes of these rare diseases not only informs patient diagnosis and management but also expands our understanding of fundamental human biology.

Interferon regulatory factors (IRFs) are a group of nine transcription factors initially identified to play a crucial role in promoting type I interferon (IFN) production (6, 7). IRFs are transiently upregulated during antimicrobial responses, where they primarily act as transcriptional mediators in IFN-induced signaling pathways (8). Beyond IFN signaling, IRFs mediate a range of immune processes, including cytokine production and the differentiation, development, and function of lymphoid and myeloid cells (9, 10, 11, 12, 13). IRF6 is unique in that it primarily mediates epithelial function and craniofacial development rather than immune responses (7, 14, 15).

IRF4, also known as MUM1, Pip, LSRIF, and ICSAT, has a vital role in immune function; however, unlike the majority of other IRFs, its primary role is not mediating IFN signaling. Instead, IRF4 is predominantly expressed in T and B cells, where it is crucial for lymphocyte development (16, 17). Multiple B cell functions rely on IRF4, including differentiation, activation, receptor editing, proliferation, class switch recombination, and plasma cell formation (9, 18, 19). Mice deficient in Irf4 exhibit impaired late-stage B cell maturation and markedly reduced serum Ig levels (9). Irf4 also plays a critical role in T cell biology, with T cell differentiation and effector fate decisions governed by graded Irf4 expression downstream of TCR signal strength (20, 21).

**Figure 1.**
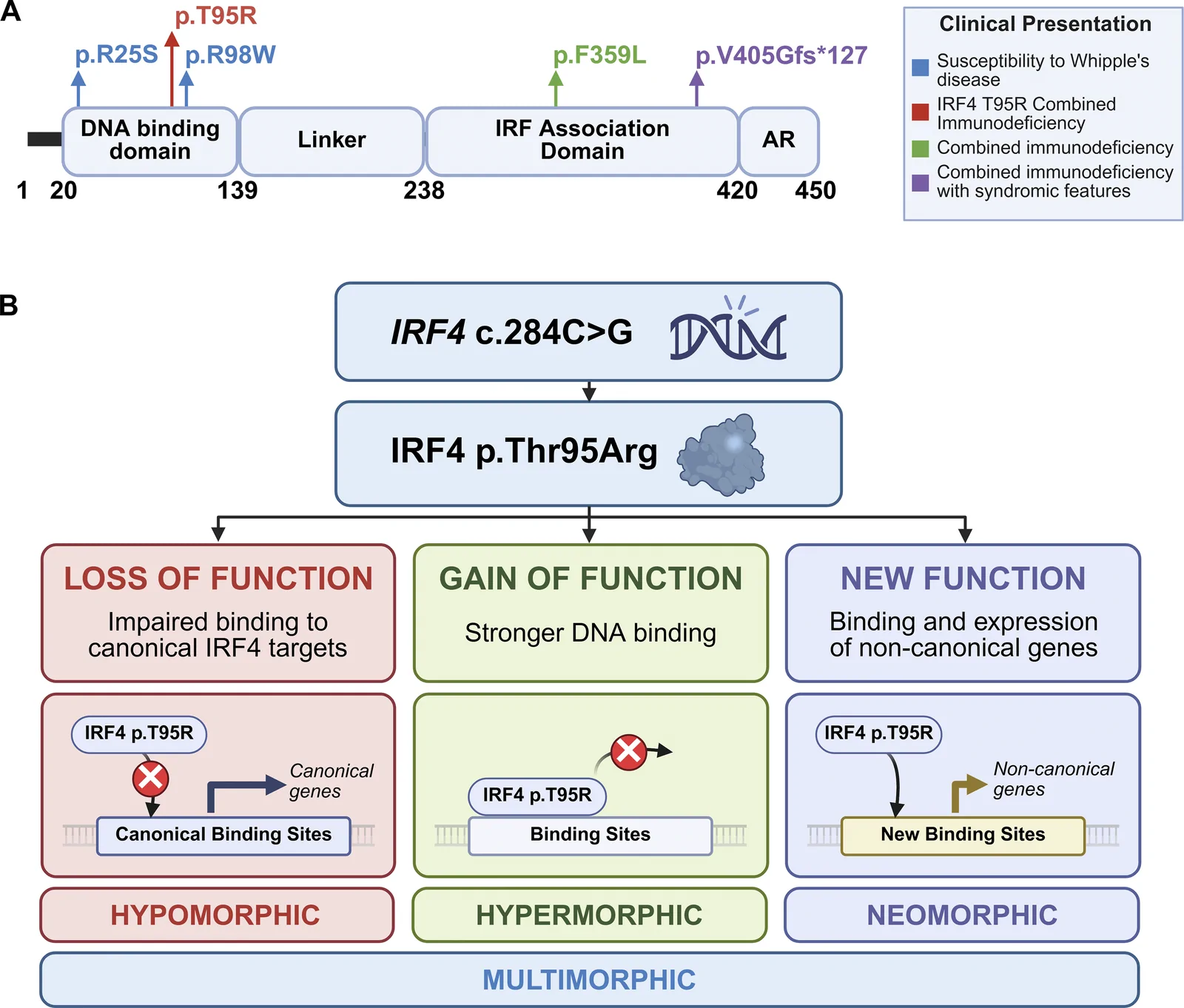
**Location and multimorphic mechanism of action of the IRF4 p.T95R** **variant. (A)** Locations of all known IEI-associated IRF4 variants color-coded according to their clinical presentation. Three are found in the DNA-binding domain, including IRF4 p.T95R, and two are located in the IRF association domain. **(B)** IRF4 p.T95R demonstrates unusual multimorphic effects: hypermorphic through increased DNA-binding affinity, hypomorphic with poor expression of canonical IRF4 target genes, and neomorphic with expression of non-canonical targets such as CXCL13. Created in BioRender. Turvey, S. (2026) https://BioRender.com/6ueehz9.

In 2023, the IRF4 International Consortium defined a novel IEI caused by a recurrent heterozygous missense variant affecting the DNA-binding domain of *IRF4* (*IRF4* NM_001195286.2 c.284C>G, p.Thr95Arg, henceforth IRF4^T95R^) (Fig. 1 A), identified in seven patients from six unrelated kindreds (22). IRF4^T95R^ patients presented with fully penetrant, early-onset, autosomal dominant combined immunodeficiency. Key clinical features included marked susceptibility to opportunistic infections, most notably *Pneumocystis jirovecii* pneumonia (PJP). In addition, patients experienced significant viral infections, including CMV and EBV, as well as mycobacterial infections. Patients had reduced CD19^+^ B cells with a prominent maturation defect, with increased naïve and transitional B cells, defective plasma cell differentiation, reduced class-switched memory B cells, and agammaglobulinemia. Furthermore, T helper 17 (Th17) and T follicular helper (T_FH_) cells were reduced with impaired IL-12 and IFN-γ production, and CD4^+^ and CD8^+^ T cells were distributed abnormally in the multidimensional space. Natural killer (NK) cells and monocytes were normal.

Biochemically, IRF4^T95R^ protein levels were normal in multiple cell types, but B cells transduced with IRF4^T95R^ demonstrated abnormal IRF4 localization, impaired class switch recombination, and reduced plasma cell differentiation. DNA-binding affinity was increased, likely due to the change to positively charged arginine, allowing more and longer interactions with DNA, resulting in an increased nuclear-to-cytoplasmic ratio. However, the expression of canonical targets was impaired, thus demonstrating simultaneous hyper- and hypomorphic effects. Furthermore, IRF4^T95R^ bound noncanonical GATA-containing sequences, upregulating a novel subset of genes, demonstrating the neomorphic effects of IRF4^T95R^. Irf4^T95R^ knock-in mice recapitulated the patient phenotype, including limited plasmablast generation, reduced IgG levels with few specific IgM and IgG antibodies, and altered T cell subsets. Collectively, IRF4^T95R^ demonstrated multimorphic behavior, with hypermorphic activity reflected by enhanced DNA binding, hypomorphic effects evidenced by diminished transcription of canonical target genes, and neomorphic activity characterized by engagement of novel target loci (Fig. 1 B).

The initial description of the IEI caused by IRF4^T95R^ focused primarily on defining the unusual multimorphic molecular mechanisms underlying disease. The present report expands the clinical understanding of the IRF4^T95R^ variant through detailed characterization of the clinical phenotype and natural history, including three additional patients not included in the original description. We also address therapeutic considerations for individuals with IRF4^T95R^, with particular emphasis on outcomes following hematopoietic stem cell transplantation (HSCT).

## Results

### IRF4^T95R^ cohort overview

To date, 10 individuals with the IRF4^T95R^ variant (NM_002460.4(IRF4):c.284C>G (p.Thr95Arg), ClinVar variation ID: 2987365, accession: VCV002987365.3) have been identified. Seven were included in the original report (22), and patients are therefore numbered according to that initial publication. A follow-up clinical report for patient 1 (P1) was recently published by Wang and colleagues, and these results have been incorporated into the present analysis (23). Patients 8, 9, and 10 (P8, P9, and P10) are newly reported in this cohort, having been identified to carry the IRF4^T95R^ variant by next-generation sequencing. P10 is also discussed by Vignesh et al. (unpublished data).

**Figure 2.**
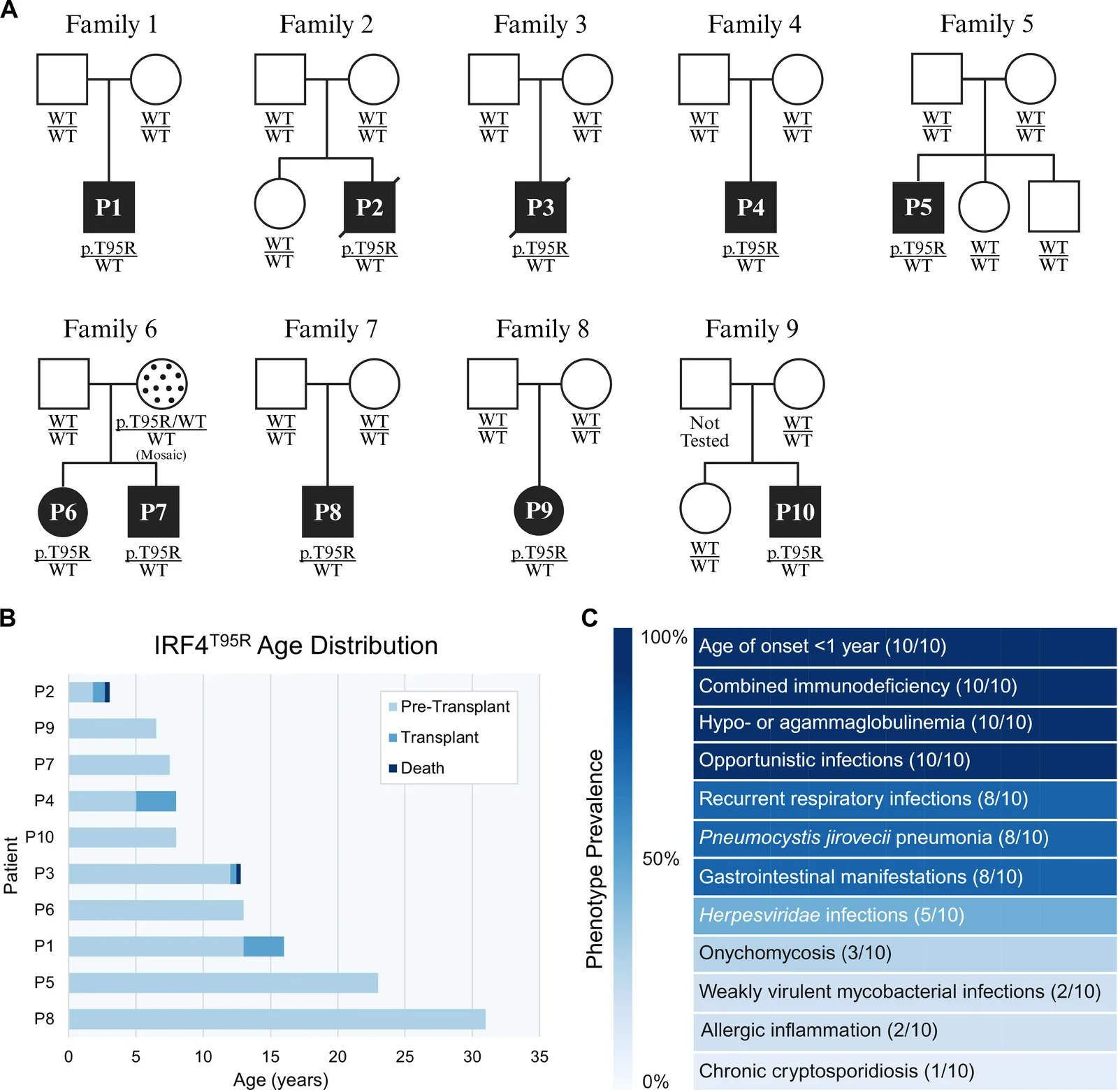
**The IRF4 p.T95R cohort and clinical manifestations. (A)** Pedigrees of the nine kindreds carrying the IRF4 p.T95R variant. The p.T95R variant was documented to have occurred de novo in all kindreds except family 6, where it was inherited from the healthy mosaic mother, and family 9, where the father’s genotype is unknown. **(B)** Age distribution at the most recent assessment or death for IRF4 p.T95R patients. **(C)** Prevalence of clinical phenotypes across the IRF4 p.T95R cohort. Created in BioRender. Turvey, S. (2026) https://BioRender.com/3hdym9o.

Seven of 10 cases are formally documented to have arisen de novo. In one family (F6), the two affected siblings inherited the IRF4^T95R^ variant from their unaffected mosaic mother, and in one family (F9), the father’s genotype is unknown (Table 1 and Fig. 2 A). Patients spanned four continents, without any evidence of a founder effect. Disease onset was uniformly early (<1 year), with a male predominance (8/10). The mean age of surviving patients at last follow-up was 14 years (range 6.5–31 years; median 10.5 years) (Fig. 2 B). Of the four patients who received HSCT, two died: P2 at 3 years of age from multidrug-resistant *Klebsiella pneumoniae* infection, and P3 at 12 years 10 mo of age from thrombotic microangiopathy-associated multi-organ failure. Notably, both had active infections at the time of transplant, with CMV and *Cryptosporidium*, respectively. One patient (P9) is awaiting transplantation. Individual patient narratives are provided in Case summaries.

**Table 1.** IRF4 p.T95R cohort

Family	Patient	Age (at last update, years)	Status	Sex	Inheritance	HSCT	Onset <1 year
F1	P1	16	Alive	Male	De novo	Yes	Yes
F2	P2	Dead (3)	Dead	Male	De novo	Yes	Yes
F3	P3	Dead (13)	Dead	Male	De novo	Yes	Yes
F4	P4	8	Alive	Male	De novo	Yes^a^	Yes
F5	P5	23	Alive	Male	De novo	No	Yes
F6	P6	13	Alive	Female	Mosaic mother	No	Yes
	P7	7.5	Alive	Male	Mosaic mother	No	Yes
F7	P8	31	Alive	Male	De novo	No	Yes
F8	P9	6.5	Alive	Female	De novo	No^b^	Yes
F9	P10	8	Alive	Male	Unknown	No	Yes

a Awaiting retransplant following graft failure.

b Awaiting first HSCT.

### Clinical and immunological manifestations

All patients with IRF4^T95R^ demonstrated marked susceptibility to opportunistic infections, involving a broad spectrum of bacterial, viral, fungal, and parasitic pathogens (Fig. 2 C and Table S1). The most frequent infection was PJP, observed in eight of 10 patients. Herpes family viral infections were documented in six of 10 patients, including CMV (3/10), HSV (1/10), EBV (1/10), and varicella-zoster virus (VZV; 1/10). Fungal infections were also notable, with oral candidiasis reported in two of 10 patients and onychomycosis in three of 10 patients. Two individuals developed mycobacterial disease, including one case of *Mycobacterium bovis* infection and one case of BCGitis following Bacillus Calmette–Guérin (BCG) vaccination. One patient experienced chronic *Cryptosporidium* infection, representing the only documented parasitic infection in this cohort.

When considering affected organ systems, respiratory manifestations were universal, with recurrent respiratory infections observed in eight of 10 patients. All patients experienced at least one episode of pneumonia, and additional respiratory findings included bronchiectasis (3/10) and airway hyperresponsiveness (2/10). Gastrointestinal (GI) manifestations were present in eight of 10 patients, including infection-associated (4/8), unclassified (3/8), graft-versus-host disease (GVHD)–associated (2/8), medication-associated (1/8), and allergy-associated (1/8) manifestations (Table S2). Diarrhea occurred in six patients (6/10) and was attributed to multiple etiologies, including food allergy, etanercept-associated toxicity, and rotavirus infection. Other GI findings included colitis (4/10, two CMV-associated), proctocolitis (1/10), mucosal eosinophilia (1/10), type B gastritis (1/10), and esophageal candidiasis (1/10). One patient (P3) developed sclerosing cholangitis secondary to chronic *Cryptosporidium* infection.

All patients exhibited severe defects in B cell development and/or antibody production, consistent with findings from the initial report, with newly identified patients demonstrating similar immunologic patterns (22) (Table S3). All individuals had either hypo- (5/10) or agammaglobulinemia (5/10), consistent with a profound impairment of B cell function. We report the immune phenotyping of two new patients not included in the original description (i.e., P8 and P9). Total CD19^+^ B cell counts were normal in P8 and P9; however, a prominent maturation defect was observed with elevated naïve B cells, low memory B cells, and very low class-switched memory B cells, consistent with findings in the initial cohort. T cells were also abnormally distributed, with low recent thymic emigrants in P8 and low Th17 and memory CD8^+^ T cells, with an elevated CD4^+^:CD8^+^ ratio in P9. NK cells were also low in P9. Detailed immunophenotyping data were unavailable for P10. Two patients (P9 and P10) in the expanded cohort presented with allergic inflammation, with P9 having eczematous eruptions beginning at 3 mo of age, and P10 having eczematous eruptions and food sensitivity beginning at 6 mo of age. Only P10 had elevated IgE levels.

All patients received IgG replacement therapy. The majority were given cotrimoxazole prophylaxis (8/10), a subset received fluconazole prophylaxis (3/10), and one was treated with azithromycin prophylaxis. All patients received antimicrobial therapy for active infections.

### HSCT in IRF4^T95R^ patients

**Figure 3.**
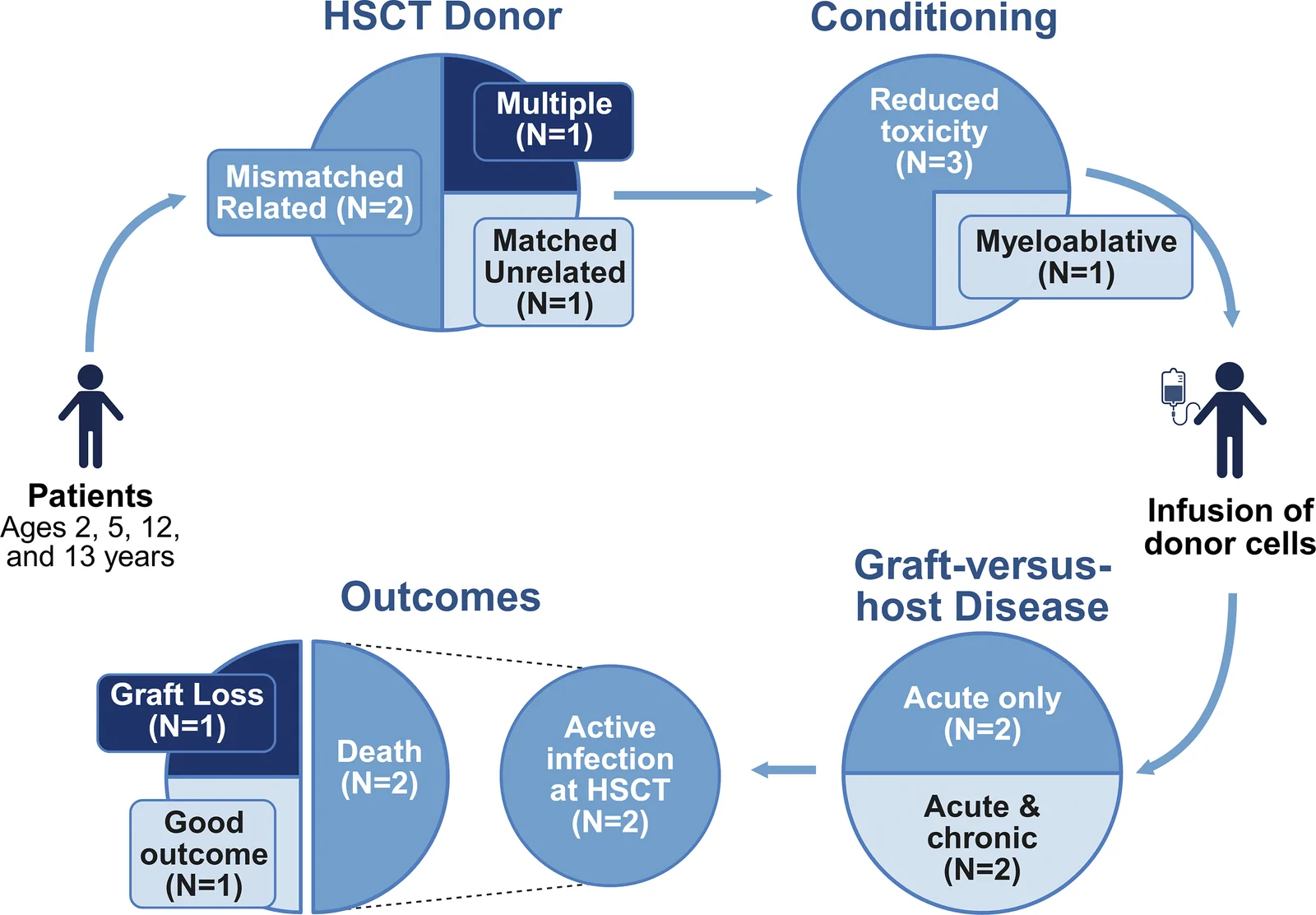
**HSCT considerations and outcomes for IRF4 p.T95R patients.** Four patients from the IRF4 p.T95R cohort were transplanted, with donors, conditioning, and immunosuppression regimens varying across the cohort. One patient had multiple HSCT donors, with the initial graft from a matched unrelated donor and subsequent grafts from the same mismatched related donor. Outcomes were mixed, with one patient having an overall good outcome, one with secondary graft failure, and two deaths occurring in patients with active CMV and *Cryptosporidium* infection, respectively, at the time of transplant. Created in BioRender. Turvey, S. (2026) https://BioRender.com/qgt16nj.

**Figure S1.**
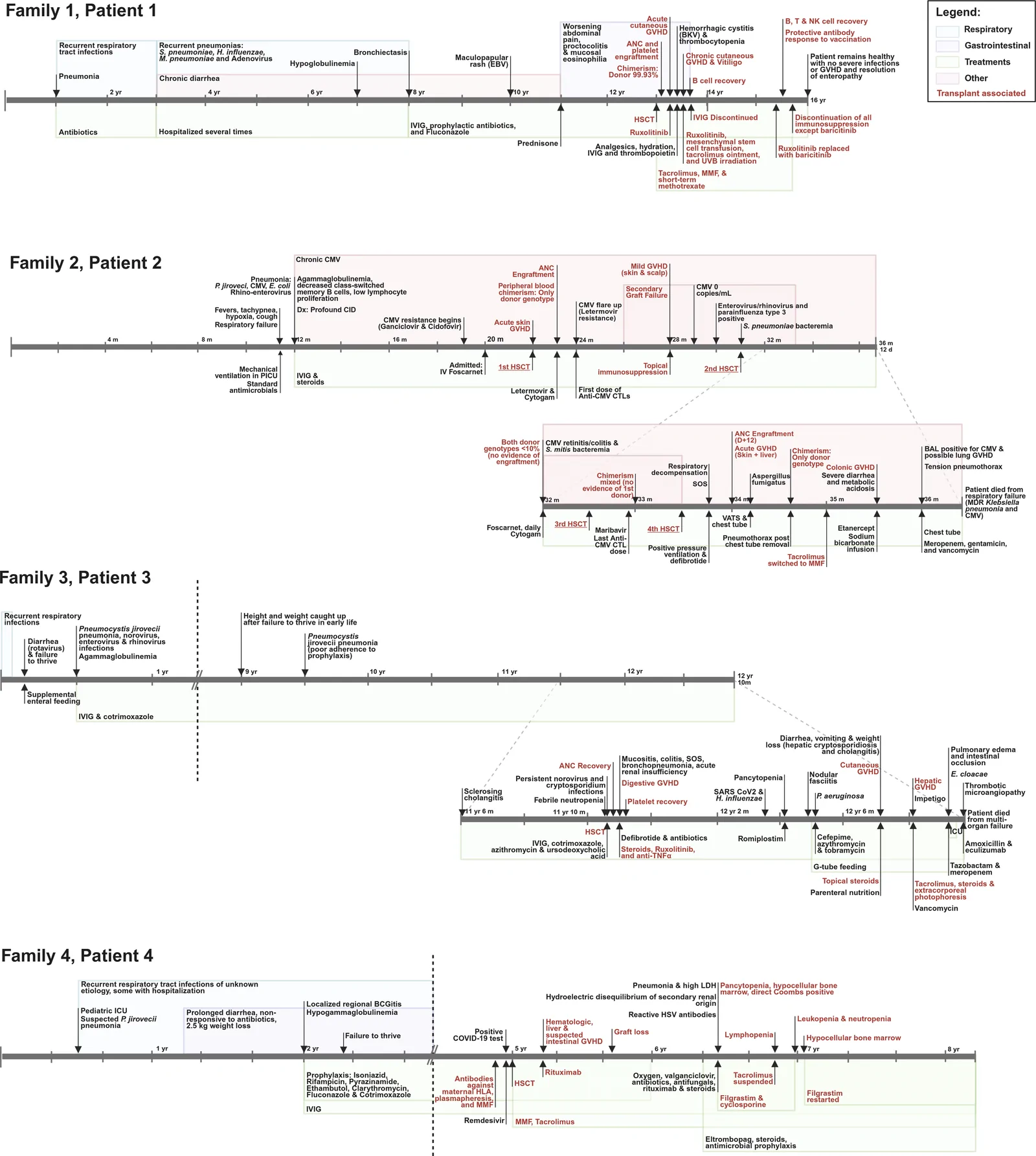
**Timelines of IRF4 p.T95R patients who received HSCTs.** Timelines for the four transplanted IRF4^T95R^ patients, from birth to last follow-up. M, month; d, day; IVIG, intravenous Ig; ANC, absolute neutrophil count; BKV, BK virus; MMF, mycophenolate mofetil; PICU, pediatric intensive care unit; CID, combined immunodeficiency; CTL, cytotoxic T lymphocytes; VATS, video-assisted thoracoscopic surgery; BAL, bronchoalveolar lavage; MDR, multidrug resistant; G-tube, gastrostomy tube; HSV, herpes simplex virus. Created in BioRender. Turvey, S. (2026) https://BioRender.com/kt56c58.

Four of 10 patients underwent HSCT at a median age of 8 years (range 2–13 years) (Table 2, Fig. 3, and Fig. S1). Two patients survived with a median follow-up of 37 mo, whereas two died at 9 and 12.5 mo following HSCT.

**Table 2.** Transplantation in IRF4 p.T95R patients

Patient	Onset (mo)	Symptoms before HSCT	Active infection at HSCT	Age at HSCT (mo)	Donor source	HLA matching	Conditioning	GVHD prophylaxis	Engraftment		Donor chimerism (max. %)	GVHD (treatment)	Outcome (follow-up, mo) complications
									ANC (days after HSCT)	Platelet (days after HSCT)			
P1	6	Hypogammaglobulinemia, recurrent pneumonia, chronic enteropathy, and EBV viremia	No	156	Fresh G-CSF PB	MUD	RTC: FLU (150 mg/m^2^), BU (0.95 mg/kg QID × 3 days), rATG (7.5 mg/kg)	TAC, MMF, MTX	12	11	>95%	aGVHD: Stage 3 skin (steroids, ruxolitinib) cGVHD: Skin (steroids, ruxolitinib, MSC transfusion, TAC ointment, and NB-UVB irradiation)	Alive (+36) BK hemorrhagic cystitis
P2	11	Agammaglobulinemia, multi-drug-resistant CMV, and hypoxic respiratory failure secondary to PJP and CMV coinfection	CMV	1st: 22	Cryopreserved G-CSF PB	MUD	RTC: TREO (42 g/m^2^), FLU (150 mg/m^2^), ALEM (1 mg/kg)	TAC, MMF	25	14	>95%	aGVHD: Stage 2 skin and liver (Steroids)	Dead (+14) Secondary GF due to drug-resistant CMV infection requiring CTL therapy
				2nd: 31 Replicate infusion: 32	TCRαβ/CD19-depleted G-CSF PB	MMRD (father)	RTC: TREO (42 g/m^2^), FLU (160 mg/m^2^), RTX, TCZ	ABT	NA	NA	11%	NA	PGF requiring reinfusion of back-up product; worsening CMV infection; bacteremia; fungemia
				3rd: 33	Cryopreserved G-CSF PB from MMRD in 2nd HSCT	MMRD (father)	RIC: FLU (30 mg/m^2^), CY (2 g/m^2^), 200 cGy TBI	TAC, MMF	12	NA	>95%	aGVHD: Stage 4 lower GI	ES, SOS, CMV pneumonitis. Died from MDR septicemia
P3	1	Agammaglobulinemia with *Cryptosporidium *and norovirus coinfection	*Cryptosporidium *and norovirus	144	TCRαβ/CD19-depleted G-CSF PB	MMRD (sister)	RTC: TREO (42 g/m^2^), FLU (160 mg/m^2^), THIO (10 mg/kg), ALEM (0.4 mg/kg), RTX	CSA	10	20	>95%	aGVHD: Stage 3/4 lower GI and liver (steroids, ruxolitinib, and anti-TNFα) cGVHD: Skin (steroids and TAC)	Dead (+9) SOS, MOF due to TA-TMA
P4	6	Hypogammaglobulinemia, recurrent respiratory infections, and prolonged diarrhea	No: Treated SARS-CoV-2 5 days before transplant	60	Fresh G-CSF PB	MMRD (mother)	MAC: BU (16 mg × 4 days), CY (60 mg/kg × 2 days), rATG (2 mg/kg × 2 days)	PT-CY, TAC, MMF	14	14	>95%	aGVHD: Stage 1 skin, stage 3 liver and possible lower GI (Rituximab)	Alive (+38) Secondary graft failure awaiting 2nd HSCT

ABT, abatacept; aGVHD, acute GVHD; ALEM, alemtuzumab; BU, busulfan; cGVHD, chronic GVDH; CSA, cyclosporine; CTL, cytotoxic T lymphocyte; CY, cyclophosphamide; ES, engraftment syndrome; FLU, fludarabine; GF, graft failure; MDR, multidrug-resistant; MMF, mycophenolate mofetil; MMRD, mismatched related donor; MOF, multiple organ failure; MSC, mesenchymal stem cell; MUD, matched unrelated donor; NA, not achieved; NB-UVB, narrow-band ultraviolet B; PB, peripheral blood; PGF, poor graft function; PT-CY, posttransplant cyclophosphamide; rATG, rabbit antithymocyte globulin; RIC, reduced intensity conditioning; RTX, rituximab; SOS, sinusoidal obstruction syndrome; TA-TMA, transplant-associated thrombotic microangiopathy; TAC, tacrolimus; TBI, total body irradiation; TCZ, tocilizumab; TREO, treosulfan.

Transplant approaches were heterogeneous, reflecting disease severity and donor availability, as well as varied local protocols. For their initial HSCT, two patients received matched unrelated donor grafts, and two underwent haploidentical related donor transplantation. One haploidentical recipient received an ex vivo TCRα/β- and CD19-depleted graft, while the other received posttransplant cyclophosphamide–based GVHD prophylaxis. Upfront conditioning regimens were primarily reduced-toxicity conditioning (RTC, 3/4), with myeloablative conditioning (MAC) in P4 (Table 2). 2/4 patients treated with RTC received treosulfan/fludarabine-based conditioning (Treo/Flu), whereas the remaining patient received a combination of treosulfan and busulfan. Salvage regimens were used in P2 following graft failure, including both RTC with Treo/Flu and reduced intensity conditioning with fludarabine, cyclophosphamide, and total body irradiation.

Engraftment kinetics were generally prompt in successful procedures, with a median time to neutrophil recovery of 13 days (range 10–25 days) and platelet recovery a median of 14 days (range 11–20 days) for the first HSCT. Although donor chimerism exceeding 95% was achieved in all transplanted patients, durable engraftment was not consistently maintained. Primary graft failure or graft loss occurred in two of four patients. P2 required multiple subsequent transplants due to recurrent graft failure, including secondary graft loss in the setting of refractory CMV infection. Bone marrow aspirate following his second HSCT was edematous and necrotic without evidence of significant hematopoietic regeneration, demonstrating that damage to the hematopoietic niche may have contributed to his repeat graft failure. P4 experienced graft loss after the first HSCT and was awaiting re-transplantation at the time of writing. These findings highlight intrinsic challenges in achieving durable engraftment in this disorder.

GVHD occurred in all transplanted patients despite prophylaxis. Acute GVHD was universal and frequently involved the skin and GI tract, with additional liver involvement. Severe manifestations included stage 4 lower GI GVHD and multi-organ involvement. One patient developed chronic GVHD with cutaneous depigmentation consistent with vitiligo (23). GVHD-directed therapies were intensive and multimodal (Table 2).

Infectious complications were frequent and clinically consequential. Both patients who ultimately died had active infections at the time of HSCT: CMV in one (P2) and *Cryptosporidium* and norovirus in the other (P3). CMV reactivation and drug-resistant CMV disease contributed to graft failure and required CMV-specific cytotoxic T lymphocyte therapy in P2. Additional complications in those undergoing HSCT included BK virus–associated hemorrhagic cystitis, bacteremia, fungemia, sinusoidal obstruction syndrome (SOS), transplant-associated thrombotic microangiopathy, and multidrug-resistant septicemia.

All deaths in this cohort occurred following HSCT and were attributed to active infections at the time of transplant. Collectively, HSCT in four IRF4^T95R^ patients was associated with high rates of graft instability, universal acute GVHD, and substantial infection-related morbidity and mortality. While immune reconstitution was achievable in selected patients, transplant-related toxicity remained a major determinant of outcome.

## Discussion

This study expands current understanding of the disease course associated with the heterozygous IRF4^T95R^ variant by defining the clinical manifestations and treatment outcomes in 10 affected patients, representing all diagnosed cases (22, 23). As such, this series provides the most comprehensive description currently available of this rare disorder.

IRF4 T95R combined immunodeficiency is defined by marked susceptibility to a broad spectrum of infections. Patients are at particularly high risk of morbidity and mortality from opportunistic and viral pathogens, including *P. jirovecii*, EBV, CMV, and *Cryptosporidium*. Laboratory investigation demonstrated evidence of combined humoral and cellular immune dysfunction (22, 24) (Table S2). B cell immunity was profoundly impaired, with very low or absent serum antibody levels, reduced total CD19^+^ B cell numbers, and a marked differentiation arrest characterized by accumulation of naïve B cells and depletion of class-switched memory B cells and plasmablasts. T cell immunity was quantitatively preserved but qualitatively abnormal, with altered subset distribution, reduced Th17 and T_FH_ populations, and impaired cytokine production, including reduced IL-2 and IFN-γ. Together, infection history and laboratory evidence of combined humoral and cellular immune dysfunction support IRF4^T95R^ causing a profound combined immunodeficiency (CID).

Clinically, patients exhibited early-onset, severe, and recurrent infections involving multiple organ systems. Respiratory disease was universal, with most patients developing pneumonia within the first year of life, frequently due to *P. jirovecii*. Additional respiratory complications included bronchiectasis and airway hyperreactivity, underscoring progressive pulmonary involvement as a defining feature. GI manifestations, a recognized component of many IEIs (25, 26), were observed in eight patients, commonly infection-associated. Notably, GI involvement was associated with an overall more severe clinical course, including progression to HSCT, whereas patients without GI disease had comparatively favorable outcomes. This interpretation is, however, limited by the small sample size and circular nature of causality, with it remaining undetermined if underlying GI dysfunction predisposed to infection, or if infection preceded GI dysfunction. It thus remains to be explored if GI dysfunction acts as an independent prognostic marker or simply a surrogate for uncontrolled infections. Finally, allergic inflammation was present in 20% of our cohort, consistent with the rate in the general population (27). Together, these findings establish IRF4^T95R^-associated disease as a CID with prominent respiratory involvement and variable but important GI disease.

Of the 10 known IRF4^T95R^ patients, only two are female (P6 and P9). Both generally exhibited a milder disease course, with neither experiencing any chronic infections, GI manifestations, or respiratory failure requiring ventilation. Ultimately, identification of additional patients will be required to determine whether biological sex modifies the clinical phenotype of IRF4 T95R.

Management of IRF4^T95R^ patients has largely relied on prevention and treatment of infection, including prophylaxis with cotrimoxazole, fluconazole, and Ig replacement therapy. Although these measures reduce infection frequency, they do not fully prevent recurrent infections or disease-related complications. Given the severity and breadth of immune dysfunction observed in these patients, HSCT should be considered on an individual basis to optimize long-term outcomes. This rationale is supported by the tissue expression pattern of IRF4, which is highly enriched in hematopoietic lineages (28). HSCT thus has the potential to restore immune function and improve survival and quality of life when appropriately tailored to patient-specific factors. The primary triggers for moving to transplant in our cohort varied, primarily centering around frequency and severity of infections. However, the observation that two patients have survived into their third and fourth decades of life without HSCT underscores that supportive care alone can confer meaningful clinical benefit in selected cases. Even with the most up-to-date treatment, patients are at lifelong risk of opportunistic infections, which can lead to significant morbidity and mortality. This highlights the need for careful, individualized risk–benefit assessment when considering HSCT.

HSCT in IRF4^T95R^-associated disease carries a substantial risk when performed in the setting of active infection. In our cohort, transplant-related mortality occurred exclusively in patients with uncontrolled infection at the time of HSCT, reinforcing the importance of timing as the most modifiable approach to improve outcomes. Successful engraftment resulted in immune reconstitution, supporting the curative potential of HSCT when undertaken before irreversible organ damage and infectious deterioration. Conditioning approaches varied by site according to patient status and local guidelines. Generally, patients who received Treo/Flu conditioning for their first graft (P2 and P3) had worse outcomes, including endothelial damage (SOS and thrombotic microangiopathy) and death, despite reduced-toxicity regimens (29). Due to our small cohort and the complex clinical status of these patients, it is not possible to determine if an alternative conditioning approach would be more suitable. While all deaths in this cohort occurred after transplant, these were the more severe cases pre-transplant, and thus those less likely to have good outcomes owing to their already fragile health status.

A notable feature of this cohort is the high rates of both graft failure and GVHD. Two patients (P2 and P4) experienced graft failure following HSCT. P2 had secondary graft failure of his first graft and primary failure of his second. Engraftment was finally achieved following the fourth HSCT. In the case of P2, active CMV and the use of third-party anti-CMV cytotoxic T lymphocytes may have been a contributing factor (30). Before transplantation, P4 was treated for anti-donor antibodies and COVID-19, which may underlie P4’s graft failure despite busulfan-based MAC. Together, graft loss in our cohort may be attributable to a wide number of factors, including underlying infection and/or inflammatory status (31, 32).

All transplanted patients were affected by GVHD, possibly influenced by pre-existing active inflammation and enteropathy at the time of transplant. We previously described upregulation of CXCL13 3–4.5 times that of healthy controls in this cohort (22). Elevated CXCL13 has been linked to GVHD in both murine models and human patients (33, 34). This may thus predispose this cohort to GVHD. Furthermore, elevated CXCL13 has also been associated with inflammatory bowel disease, showing a positive correlation with inflammation severity, further supported by CXCL13 knockout mice that demonstrated reduced colitis severity (35, 36). It is plausible that elevated CXCL13 could contribute indirectly to the apparent engraftment defect in our cohort. Other biomarkers, such as ferritin and type I and II IFNs, could provide further insight. In our cohort, ferritin was normal for all patients assessed, and type II IFNs were reduced; however, type I IFNs were not assessed (22). Further work may benefit from the analysis of a range of biomarkers in this patient population, particularly before and after transplant.

**Figure 4.**
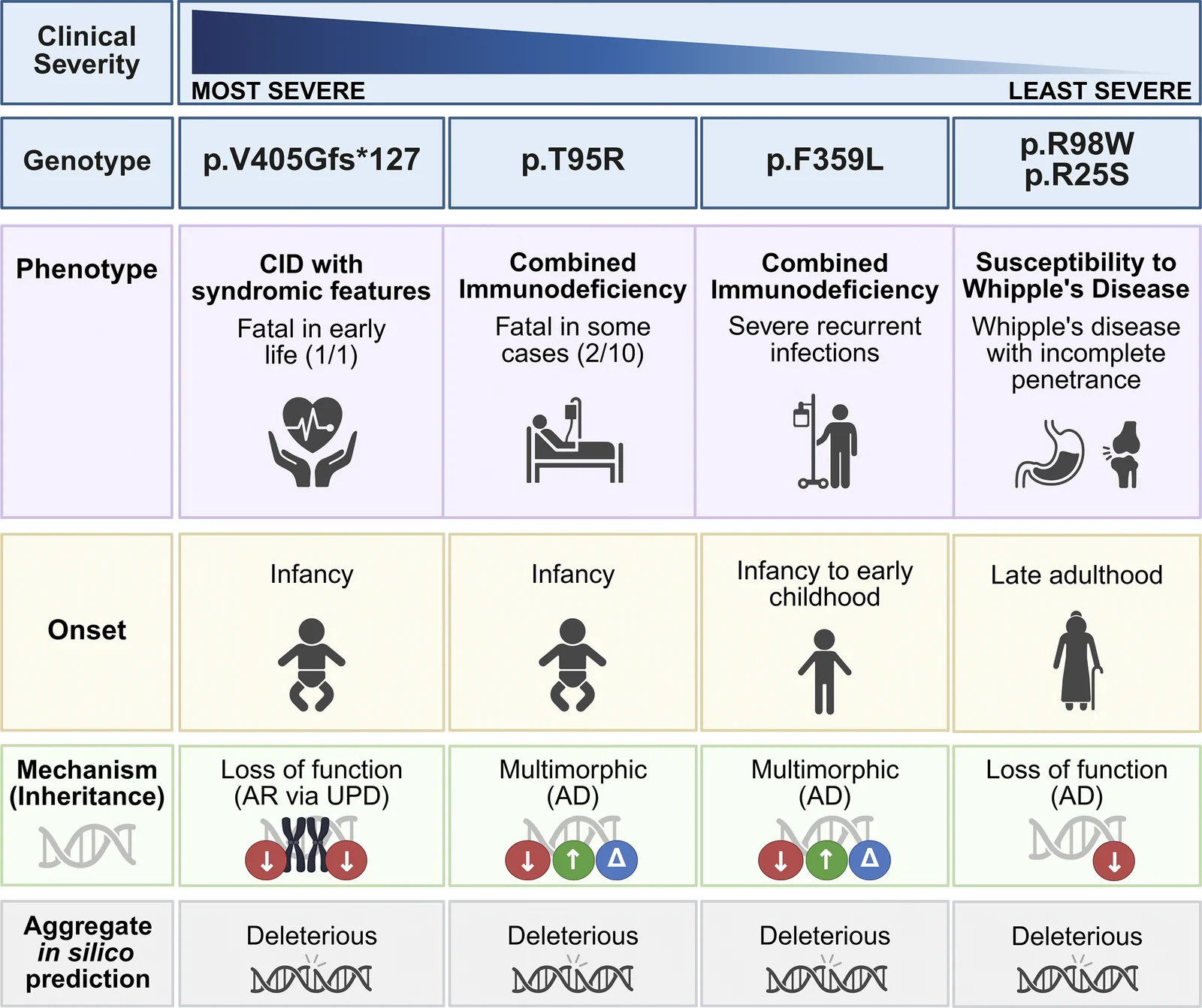
**The clinical spectrum of *IRF4*-associated IEIs.** Genotypes, phenotypes, onset, and mechanisms of known *IRF4*-associated IEIs are presented (22, 37, 38, 39, 40). An aggregate in silico score was calculated based on the majority prediction across CADD, AlphaMissense, REVEL, and SpliceAI models. Full in silico results are available in Table S4. CID, combined immunodeficiency; AR, autosomal recessive; UPD, uniparental disomy; AD, autosomal dominant. Created in BioRender. Turvey, S. (2026) https://BioRender.com/bahcqv2.

To date, multiple distinct IEIs affecting *IRF4* have been described, each characterized by different genetic lesions, inheritance patterns, and spectra of infectious susceptibility (7) (Fig. 4 and Table S4). Accordingly, IEIs involving *IRF4* should be considered as discrete clinical and molecular entities rather than grouped under the imprecise and potentially misleading label of “IRF4 deficiency.”

The first reported *IRF4*-associated IEI was autosomal dominant susceptibility to Whipple’s disease, caused by a missense *IRF4* variant (p.R98W) (38). This paradigm was subsequently reinforced by the description of Whipple’s disease in a second family carrying a distinct missense *IRF4* variant (p.R25S) (40). Another unique case was an *IRF4*-associated IEI involving an infant with combined immunodeficiency inherited through uniparental disomy of chromosome 6, resulting in homozygosity for a frameshift *IRF4* variant (p.V405GfsTer127) (37). However, this complex genetic context makes it difficult to definitively attribute the phenotype to the *IRF4* genotype alone. More recently, two heterozygous missense variants in *IRF4*, p.T95R and p.F359L, were shown to cause autosomal dominant disease through multimorphic mechanisms (22, 39). Importantly, the clinical consequences of these variants differ markedly. In contrast to the profound combined immunodeficiency caused by IRF4^T95R^, IRF4^F359L^ is associated with a milder phenotype without reported fatalities, causing recurrent infections managed with supportive care. At a mechanistic level, both variants exhibit multimorphic behavior, but in distinct ways, highlighting variant-specific rewiring of IRF4-dependent transcriptional programs.

Together, the spectrum of pathogenic *IRF4* variants described to date constitutes an allelic series in which distinct genetic lesions give rise to mechanistically and clinically divergent immune phenotypes. IRF4^T95R^ represents the most severe end, defining a distinct CID entity rather than a variant of previously reported *IRF4*-associated disorders. Clinicians encountering patients with missense variants in *IRF4* should avoid reflex categorization under a single diagnostic or therapeutic framework and instead pursue variant-specific interpretation that integrates functional data, inheritance pattern, and clinical severity. Notably, multimorphic IRF4 variants appear to have more severe phenotypes than loss-of-function variants, supporting the value of wet-lab assessment when possible. These considerations are essential for prognostication and for guiding management decisions, including whether supportive care alone is appropriate or whether HSCT should be considered. Ultimately, identification of additional patients with distinct *IRF4* variants will be necessary to refine genotype–phenotype correlations and inform clinical care.

This study has both strengths and limitations. Although we have aggregated data from all currently identified IRF4^T95R^ patients worldwide, our findings are necessarily limited by the small sample size of ten affected individuals. Despite this constraint, this work represents the first comprehensive report focused on the clinical course, outcomes, and management considerations of IRF4^T95R^-associated disease, and we anticipate that these observations will inform the diagnosis, clinical monitoring, and therapeutic decision-making for future patients.

In summary, this study defines IRF4^T95R^ as a distinct IEI characterized by profound combined immunodeficiency, severe early-onset infections, and substantial clinical heterogeneity. These findings have immediate implications for variant interpretation, prognostication, and clinical management, including consideration of HSCT, which has the potential to be curative but is most likely to be effective when undertaken early, before the development of advanced or uncontrolled infections. Ultimately, identification of additional patients and variants will be essential to refine genotype–phenotype correlations, guide individualized treatment decisions, and further elucidate the role of IRF4 in human immune regulation.

## Materials and methods

All study participants and/or their parents/guardians provided written informed consent for sequencing, data analysis, and publication of findings. Research study protocols were approved by local institutions, specifically the University of British Columbia Clinical Research Ethics Board (H15-00641 and H18-02853); Comité de Protection des Personnes Ile de France II, Paris, France (reference: 2015-01-05; 2015-01-05 MS2); the French Advisory Committee on Data Processing in Medical Research (Comité Consultatif sur le Traitement de l’information en matière de Recherche dans le domaine de la Santé, Paris, France; reference: 15.297bis); the Vanderbilt University Medical Center Human Research Protection Program (Institutional Review Board no.: 200412); the University of Ulm ethics board, Germany (application number 156/11); and the National Institute of Allergy and Infectious Diseases, National Institutes of Health.

All patients were identified by clinical teams to carry the IRF4 p.Thr95Arg variant through next-generation sequencing (NM_002460.4(IRF4):c.284C>G (p.Thr95Arg), ClinVar variation ID: 2987365; accession: VCV002987365.3). Biochemical workup of P1 to P7 was completed previously (22). Comprehensive clinical reports were analyzed to track clinical characteristics associated with each case. Clinical diagnoses were performed according to local guidelines. Classification of conditioning regimens was performed according to the EMBT handbook (41). Bioinformatics analysis of previously reported IRF4 variants was performed with Franklin by Genoox (https://franklin.genoox.com).

### Case summaries

#### Family 1, P1

P1 was initially reported by the IRF4 International Consortium, with a follow-up recently published by Wang et al. (22, 23). The published findings of this case are briefly summarized here.

P1 was born to healthy nonconsanguineous parents and tolerated all pediatric vaccines, including BCG. Symptom onset was pneumonia at 6 mo, followed by repeated upper respiratory tract infections from 6 mo to 3 years. From age 3–8 years, he continued to have severe recurrent pneumonias*,* as well as bronchiectasis. At 7 years old, hypogammaglobulinemia with reduced B cells was diagnosed. IgG replacement therapy and prophylactic antimicrobials successfully reduced the frequency of respiratory infections. GI manifestations began at age 3 years with chronic diarrhea and led to worsening abdominal pain from age 11 years onward that partially resolved with prednisone. At age 10 years, P1 developed a non-itchy maculopapular rash on his face, limbs, hands, and feet and tested positive for EBV in peripheral blood.

The decision was made to move to HSCT due to the recurrent infections despite conventional therapies. P1 was grafted from a matched unrelated donor at age 13 years. Platelet and neutrophil recovery were achieved at 11 and 12 days after HSCT, respectively. 25 days after HSCT, steroid-refractory grade II acute cutaneous GVHD was diagnosed, which resolved with ruxolitinib. Late-onset hemorrhagic cystitis was attributed to polyomavirus BK. At 80 days after HSCT, P1 presented with thrombocytopenia, successfully treated with intravenous immunoglobulin (IVIG) and thrombopoietin. At 6 mo after HSCT, he presented with chronic cutaneous GVHD and began steroid therapy, with GVHD resolving within 2 mo, but vitiligo depigmentation gradually developing on the face, limbs, and trunk. IVIG replacement therapy was discontinued at 9 mo after HSCT following B cell recovery, and complete donor chimerism was sustained over the 1-year follow-up period. T, B, and NK cells normalized to age-appropriate reference ranges by 30 mo after HSCT. A protective antibody response to hepatitis B vaccination was achieved after HSCT. All immunosuppressants were discontinued by 32 mo after HSCT, except baricitinib, which replaced ruxolitinib at 28 mo after HSCT. As of the last report at 36 mo after HSCT, P1 continued on baricitinib and has remained free of infections and GVHD (23).

#### Family 2, P2

P2 was born to nonconsanguineous parents, healthy apart from a maternal history of rheumatoid arthritis. He tolerated all routine immunizations and was otherwise healthy until 11 mo of age, when he presented with respiratory failure requiring mechanical ventilation. Rhinovirus/enterovirus infection, PJP, CMV viremia, and *Escherichia coli* ventilator-associated pneumonia ensued. Immunological assessment revealed agammaglobulinemia, low class-switched memory B cells, an undetectable response to vaccination, and reduced lymphocyte proliferation. After treatment of his infections, P2 was started on a regimen of IVIG, cotrimoxazole and valganciclovir prophylaxis. P2 then moved to HSCT due to the overall clinical picture of CID with multidrug-resistant CMV viremia. At 22 mo, he received his first HSCT from a matched unrelated donor, with GVHD prophylaxis of tacrolimus and mycophenolate mofetil (MMF). He received his second graft at 31 mo, followed by a replicate infusion at 32 mo. Due to the inability to procure more transplants from the original donor, both were sourced from the haploidentical father with TCR α-β and CD19 depletion. HSCT was repeatedly complicated by graft loss due to ongoing multidrug-resistant CMV and CMV–end organ disease.

Since our last report, P2 received his third HSCT at age 33 mo. Respiratory decompensation, SOS, and cutaneous and hepatic GVHD developed. He was treated with positive pressure ventilation, video-assisted thoracoscopic surgery for an abscess of the right lower lung, and a chest tube. Microbiology was positive for *Aspergillus fumigatus,* and pathology showed chronic pneumonitis and bronchopneumonia of multifactorial etiology. Chest tube removal 1 mo later was complicated by pneumothorax. Further assessment revealed colonic grade 1 GVHD, possible lung GVHD, and bronchoscopy positive for CMV and rhinovirus/enterovirus. P2 also experienced increased diarrhea, resulting in severe metabolic acidosis requiring sodium bicarbonate infusion. Shortly thereafter, the pneumothorax worsened, and the chest tube was reinserted. Extubation was attempted unsuccessfully. The right lower lung showed evidence of bronchopleural fistula and pneumothorax with pulmonary artery hypertension. Persistent hepatomegaly and nephromegaly were observed, and a GI bleed developed. The patient’s respiratory status began an acute deterioration, where the patient could not be ventilated despite maximal settings. Chest X-ray showed interval worsening of aeration with opacification of both lungs, extensive pneumomediastinum, and persistence of right-sided pneumothorax. Blood culture showed multidrug-resistant *K. pneumoniae*. P2 died of respiratory failure at 36 mo 12 days.

#### Family 3, P3

P3 was born to healthy, nonconsanguineous parents. He presented at the age of 1 mo with bronchiolitis, recurrent respiratory tract infections, and rotavirus-associated diarrhea, requiring supplemental enteral feeding. At the age of 6 mo, he suffered from PJP, norovirus, enterovirus, and rhinovirus infections. Immunological workup revealed agammaglobulinemia with reduced memory B cells and increased naïve and reduced memory CD8^+^ T cells. IVIG and cotrimoxazole prophylaxis were started. P3’s growth caught up in the following years, and he had no significant health problems until age 9.5 years, when he suffered a second episode of PJP due to poor adherence to cotrimoxazole prophylaxis.

Since our initial report, P3 developed sclerosing cholangitis due to cryptosporidiosis at age 11.5 years, leading to the decision to move to HSCT. He was transplanted at 11 years 10 mo from his haploidentical sister with TCRαβ and CD19 depletion. He then developed mucositis, colitis, SOS, and bronchopneumonia. Despite cyclosporin prophylaxis, GI GVHD was confirmed 16 days after HSCT. absolute neutrophil count and platelet recovery were achieved at 10 and 20 days after HSCT, respectively. At 150 days after HSCT, P3 was readmitted for SARS-CoV-2 and *Haemophilus influenzae* pneumonia, and was treated for thrombocytopenia. 21 days later, P3 was hospitalized for fever, cough, vomiting, and persistent cholestasis. A benign myofibroblastic lesion was found incidentally, consistent with intramuscular nodular fasciitis. At 199 days after HSCT, a G-tube was placed. *Pseudomonas aeruginosa* was identified at 209 days after HSCT. P3 was rehospitalized at 263 days after HSCT for dehydration and weight loss resulting from ongoing cryptosporidiosis infection, including hepatic cryptosporidiosis and cholangitis, and was started on parenteral nutrition. Cutaneous and hepatic GVHD and impetigo were later confirmed and treated. At 311 days after HSCT, P3 started extracorporeal photopheresis. At 333 days after HSCT, he was transferred to the intensive care unit for intestinal occlusion, acute renal failure, and pulmonary edema, and *Enterobacter cloacae* bacteria were found in sputum. P3 died at 12 years 9 mo, 347 days after HSCT, from multi-organ failure due to thrombotic microangiopathy.

#### Family 4, P4

P4 was healthy until the age of 6 mo when he was admitted to intensive care for suspected PJP. Recurrent respiratory infections continued in early life, of unknown etiology, with some requiring hospitalization. Prolonged diarrhea began at 15 mo old, which was unresponsive to antibiotics and resulted in 2.5 kg of weight loss. At age 2 years, he developed localized BCGitis unresponsive to antibiotics. Immunological findings revealed hypogammaglobulinemia, for which prophylactic fluconazole, cotrimoxazole, and IgG replacement therapy were started. The decision was made to transplant due to continuous infectious processes despite prophylactic antibiotics and Ig replacement therapy. At age 4 years, P4 was found to have antibodies against maternal HLA and underwent two sessions of plasmapheresis and treatment with MMF in preparation for HSCT. 5 days before the planned transplant, he tested positive for SARS-CoV-2 and was treated with remdesivir. At age 5 years, P4 underwent HSCT from his haploidentical mother.

Since our initial report, P4 has had hematologic and hepatic GVHD and suspected intestinal GVHD. At 8 mo after HSCT, P4 lost his graft. At age 6 years, prophylactic treatment was paused for 2 mo in the absence of the primary clinician. Soon after, P4 presented with pneumonia, pancytopenia, and high lactate dehydrogenase. Hypocellularity was confirmed in the bone marrow, suspected to be aplastic anemia. Renal disequilibrium, secondary to antifungal therapy, was treated with oral potassium. Over the next 4 mo, varying levels of immunosuppression and filgrastim were administered to manage neutrophil and platelet counts. As of the last update, P4 is awaiting a suitable donor for a second HSCT.

#### Family 5, patient 5

P5 presented at the age of 4 mo with recurrent respiratory infections. At age 7 mo, he was admitted for PJP and respiratory CMV and recovered well after treatment. At this time, IgG, IgM, and IgA were absent, and he failed to respond to vaccination, leading to suspicion of CID. Immunophenotyping revealed normal cell counts, but a functional defect in anti-CMV T cell function. P5 was started on prophylactic cotrimoxazole and IVIG. He experienced a second bout of PJP at age 20 mo following discontinuation of cotrimoxazole. This episode required mechanical ventilation and re-initiation of cotrimoxazole. Since the age of 3, P5 has had persistent onychomycosis of the fingers and toenails.

In the following years, he had no serious complications or organ damage until a hospitalization at age 23 years for acute gastroenteritis, with severe weight loss and a clinical picture of subileus. Norovirus and enteropathogenic *E. coli* were found in his stools, and endoscopy revealed colitis, type B gastritis, and *Candida* esophagitis. A lung computed tomography also revealed atypical pneumonia with bronchiectasis. As of the last update, he is clinically well, continuing on IVIG and cotrimoxazole prophylaxis.

#### Family 6, P6 and P7

P6 and P7 were born to healthy, nonconsanguineous parents. Both siblings were vaccinated at birth, including BCG, with no adverse effects. The older sister, P6, was healthy until age 5 mo when she required hospitalization for recurrent hypoxia. She was later readmitted at 6 mo with suspected PJP, which responded rapidly to cotrimoxazole. Since early childhood, she has continued to experience repeated upper sinopulmonary infections, including ear infections, adenoiditis, and sinusitis. At age 1 year, immunological investigation found agammaglobulinemia with B cells present. At age 8 years, adenoidectomy was performed due to severe adenoid hypertrophy, and bilateral tympanostomy tubes were placed. Since our initial report, P6 has continued to have repeated adenoiditis requiring antibiotic treatment. At age 10 years, her brother (P7) tested positive for *M. bovis,* and she was treated preventively. At age 11 years, she had pneumonia of the right lung and onychomycosis with negative fungal cultures.

P7 was born 5 years after his sister P6. He was healthy until the age of 5 mo when recurrent episodes of bronchospasms began. At 7 mo, he was admitted to the hospital for PJP, which was responsive to cotrimoxazole therapy. Immunological findings at this time led to a diagnosis of agammaglobulinemia with reduced B lymphocytes. Tympanostomy tubes were placed bilaterally at age 4 years and replaced at age 6.5 years. A tonsillectomy was also performed at 4 years of age due to severe tonsillar hypertrophy. Shortly thereafter, a fast-growing cervical lymph node was also excised, and *M. bovis* was identified and treated. Since the last report, P7 presented with persistent diarrhea and weight loss. Following colonoscopy and reactive CMV PCR, he was diagnosed with infectious vs. inflammatory bowel disease, unclassified colitis, treated with ganciclovir for 21 days, then prednisone 1 mg/kg/day, with improvement. He has also had sporadic bronchospasms and onychomycosis with negative fungal cultures. Both P6 and P7 continued with Ig replacement therapy and cotrimoxazole prophylaxis.

#### Family 7, P8

P8 is new to this study, born at 38 wk of gestation due to preeclampsia to otherwise healthy, nonconsanguineous parents. Symptom onset was at 5 mo, when he presented with severe VZV infection that was responsive to standard antivirals. Immunological workup at this time showed a rich non-blastic bone marrow with a maturation block at the promyelocytic stage. B cell counts were normal, but profound hypogammaglobulinemia was observed. T cells were normal, but NK cells were absent. P8 also had neutropenia, complicated by perianal lesions that responded to GM-CSF treatment. At the same time, P8 had disseminated microvesicular lesions compatible with HSV infection, which improved with acyclovir treatment. At 8 mo old, P8 had pneumonia, which responded to antibiotics, and IVIG prophylaxis was initiated. Shortly thereafter, P8 was admitted for severe PJP, requiring mechanical ventilation. Prophylactic cotrimoxazole was continued. Microvesicles also recurred with splenomegaly, which responded to acyclovir treatment. At age 2, tonsillar, splenic, and lymphatic hyperplasia were observed.

The following years were uneventful until the age of 13 years, when P8 had an appendectomy. IVIG was discontinued at age 14. Shortly thereafter, P8 experienced repeated ear infections with persistent tonsillar hyperplasia. Cotrimoxazole was switched to azithromycin; however, PJP recurred at age 17 years, and cotrimoxazole and IVIG were resumed. P8 had a poor response to tetanus vaccination. Systematic assessment revealed rectal pseudopolyps on colonoscopy. At age 18 years, P8 had another episode of pneumonia and recurrent ear infections, which responded to antibiotic treatment. A year later, P8 had acute bronchitis, which was also responsive. The following 12 years were uneventful with the continuation of prophylactic cotrimoxazole and IVIG.

#### Family 8, P9

P9 is new to this cohort and is also discussed by Vignesh et al. (unpublished data). She was otherwise healthy until 2.5 mo of age when she presented with recurrent bacterial adenitis, impetigo, and superficial skin infections with *Staphylococcus aureus*. She also had recurrent intertrigo and oral thrush requiring fluconazole therapy. At age 4 mo, P9 was diagnosed with hypogammaglobulinemia. By age 2 years, she was found to have hyperreactive airway disease and prescribed budesonide and salbutamol inhalers. Immunological workup revealed low NK, Th17, and CD8^+^ T cells with expansion of CD4^+^ T cells; however, proliferation to PHA was normal. At this time, P9 was started on 6 mo of IVIG. At age 3 years, P9 was admitted to the hospital for severe PJP. Bronchoalveolar lavage was positive for *S. aureus* and *Candida *sp., and monthly IVIG was restarted. Despite prophylaxis, she continued to have intermittent superficial skin infections, oral candidiasis, and eczematoid eruptions. Corticosteroids were able to successfully manage the eczematoid eruptions. P9 had another episode of bacterial adenitis with *S. aureus* at age 5 years. As of the last update at age 6.5 years, P9 has ongoing recurrent infections despite antimicrobial prophylaxis and is thus being prepared for an HSCT.

#### Family 9, P10

P10 is also new to this cohort. He was born to healthy nonconsanguineous parents. Symptom onset was at 6 mo, with eczematous skin lesions on the face, chest, and upper limbs. At 9 mo, P10 was hospitalized for pneumonia requiring intensive care and discharged with home oxygen therapy. He continued to have repeated infections early in life, with white lesions in the oral cavity since age 1 year and acute sinusitis four times per year since age 3 years. Repeated nasal obstructions also began at age 3 years, with a runny nose, sneezing, and itching. At 4 years, P10 presented with a submandibular abscess of odontogenic origin, identified as penicillin-resistant *S. aureus*. Physical exam revealed macular-papular, scaly skin lesions disseminated over the chest and face with a boil-like lesion on the scalp. At 5 years of age, hypogammaglobulinemia with elevated IgE and normal B lymphocytes was documented. P10 was started on monthly Ig and azithromycin prophylaxis. Positive radioallergosorbent test (RAST) results were received for a range of food allergens (highest result for soybean: 3.99 UI/ml, RAST 3), with nonreactive results for respiratory allergens. P10 is currently aged 8 years, continuing Ig replacement and antibiotic prophylaxis, and has atopic dermatitis under treatment and food allergy under follow-up.

### Online supplemental material

Fig. S1 provides timelines for all transplanted IRF4^T95R^ patients, from birth to last follow-up. Table S1 describes the pretransplant infection susceptibility, and Table S2 describes the GI manifestations in this cohort. Table S3 presents immunophenotyping data for P8–10. Table S4 summarizes clinical characteristics and in silico scores of known IRF4 variants.

## Supplementary Material

Table S1 shows pretransplant infection susceptibility.

Table S2 shows GI manifestations in IRF4 T95R patients.

Table S3 shows immunophenotyping data of P8–10.

Table S4 shows phenotypic and in silico characteristics of known damaging IRF4 variants.

## Data Availability

Potentially identifying data cannot be shared, but de-identified data are available upon reasonable request.
