# Supplementary material for: IRF4 p.T95R combined immunodeficiency: Clinical features and transplant outcomes
Source: J Hum Immun. 2026 Oct 9;2(6):e20260069. doi: 10.70962/jhi.20260069 (PMC13651748; doi:10.70962/jhi.20260069)
Supplement: Supplementary file 1 [file jhi_20260069_tables1.docx]

| **Pre-Transplant Infection History** | | | | | | | | | | | |
| --- | --- | --- | --- | --- | --- | --- | --- | --- | --- | --- | --- |
| **Patient** | **P1** | **P2** | **P3** | **P4** | **P5** | **P6** | **P7** | **P8** | **P9** | **P10** | **Count** |
| Opportunistic infections |  |  |  |  |  |  |  |  |  |  | 10 |
| Viral Infection(s) |  |  |  |  |  |  |  |  |  |  | 7 |
| Herpes family viruses |  |  |  |  |  |  |  |  |  |  | 5 |
| Cytomegalovirus |  |  |  |  |  |  |  |  |  |  | 3 |
| Epstein-Barr virus |  |  |  |  |  |  |  |  |  |  | 1 |
| Herpes simplex virus |  |  |  |  |  |  |  |  |  |  | 1 |
| Varicella-zoster virus |  |  |  |  |  |  |  |  |  |  | 1 |
| Enterovirus |  |  |  |  |  |  |  |  |  |  | 2 |
| Rhinovirus |  |  |  |  |  |  |  |  |  |  | 2 |
| Norovirus |  |  |  |  |  |  |  |  |  |  | 2 |
| Adenovirus |  |  |  |  |  |  |  |  |  |  | 1 |
| Rotavirus |  |  |  |  |  |  |  |  |  |  | 1 |
| SARS-CoV-2 |  |  |  |  |  |  |  |  |  |  | 1 |
| Bacterial Infection(s) |  |  |  |  |  |  |  |  |  |  | 7 |
| Mycobacteria |  |  |  |  |  |  |  |  |  |  | 2 |
| Bacillus Calmette-Guérin (BCG) |  |  |  |  |  |  |  |  |  |  | 1 |
| *Mycobacterium* *bovis* |  |  |  |  |  |  |  |  |  |  | 1 |
| *Helicobacter pylori* |  |  |  |  |  |  |  |  |  |  | 2 |
| *Escherichia coli* |  |  |  |  |  |  |  |  |  |  | 2 |
| *Staphylococcus aureus* |  |  |  |  |  |  |  |  |  |  | 2 |
| *Haemophilus influenzae* |  |  |  |  |  |  |  |  |  |  | 1 |
| *Moraxella catarrhalis* |  |  |  |  |  |  |  |  |  |  | 1 |
| *Mycoplasma pneumoniae* |  |  |  |  |  |  |  |  |  |  | 1 |
| *Streptococcus pneumoniae* |  |  |  |  |  |  |  |  |  |  | 1 |
| *Staphylococcus epidermidis* |  |  |  |  |  |  |  |  |  |  | 1 |
| Fungal/Parasitic Infection(s) |  |  |  |  |  |  |  |  |  |  | 9 |
| *Pneumocystis jirovecii* |  |  |  |  |  |  |  |  |  |  | 8 |
| Onychomycosis, unspecified |  |  |  |  |  |  |  |  |  |  | 3 |
| *Candida species* |  |  |  |  |  |  |  |  |  |  | 3 |
| *Candida, unspecified* |  |  |  |  |  |  |  |  |  |  | 2 |
| *Candida albicans* |  |  |  |  |  |  |  |  |  |  | 1 |
| *Cryptosporidium* |  |  |  |  |  |  |  |  |  |  | 1 |
| No Causative Pathogen Identified |  |  |  |  |  |  |  |  |  |  | 4 |
| Pneumonia |  |  |  |  |  |  |  |  |  |  | 3 |
| Suppurative otitis media |  |  |  |  |  |  |  |  |  |  | 1 |
| Urinary tract infection |  |  |  |  |  |  |  |  |  |  | 1 |
| Ear infection |  |  |  |  |  |  |  |  |  |  | 1 |
| Impetigo |  |  |  |  |  |  |  |  |  |  | 1 |
| Acute sinusitis |  |  |  |  |  |  |  |  |  |  | 1 |

## Supplemental Table 1: Pre-transplant infection susceptibility.
