# Supplementary material for: IRF4 p.T95R combined immunodeficiency: Clinical features and transplant outcomes
Source: J Hum Immun. 2026 Oct 9;2(6):e20260069. doi: 10.70962/jhi.20260069 (PMC13651748; doi:10.70962/jhi.20260069)
Supplement: Supplementary file 2 [file jhi_20260069_tables2.docx]

| **Gastrointestinal Manifestations** | | | | | | | | | | | |
| --- | --- | --- | --- | --- | --- | --- | --- | --- | --- | --- | --- |
| **Patient** | **P1** | **P2** | **P3** | **P4** | **P5** | **P6** | **P7** | **P8** | **P9** | **P10** | **Count** |
| GI Manifestations |  |  |  |  |  |  |  |  |  |  | 8 |
| Infection-associated |  |  |  |  |  |  |  |  |  |  | 4 |
| Unclassified |  |  |  |  |  |  |  |  |  |  | 3 |
| GVHD-associated |  |  |  |  |  |  |  |  |  |  | 2 |
| Medication-associated |  |  |  |  |  |  |  |  |  |  | 1 |
| Allergy-associated |  |  |  |  |  |  |  |  |  |  | 1 |
| Diarrhea |  |  |  |  |  |  |  |  |  |  | 6 |
| Colitis |  |  |  |  |  |  |  |  |  |  | 4 |
| Proctocolitis |  |  |  |  |  |  |  |  |  |  | 1 |
| Mucosal eosinophilia |  |  |  |  |  |  |  |  |  |  | 1 |
| Sclerosing cholangitis |  |  |  |  |  |  |  |  |  |  | 1 |
| Perianal lesions |  |  |  |  |  |  |  |  |  |  | 1 |
| Rectal pseudopolyps |  |  |  |  |  |  |  |  |  |  | 1 |

**Supplemental Table 2: Gastrointestinal manifestations in IRF4 T95R patients.**
