# Supplementary material for: IRF4 p.T95R combined immunodeficiency: Clinical features and transplant outcomes
Source: J Hum Immun. 2026 Oct 9;2(6):e20260069. doi: 10.70962/jhi.20260069 (PMC13651748; doi:10.70962/jhi.20260069)
Supplement: Supplementary file 3 [file jhi_20260069_tables3.docx]

| Patient 8 | | | | | | | | | | | | | | | |
| --- | --- | --- | --- | --- | --- | --- | --- | --- | --- | --- | --- | --- | --- | --- | --- |
| Age (years) | **17** | | **Ref.** | **17** | **Ref.** | **18** | **Ref.** | **24** | **Ref.** | **30** | **Ref.** | **30** | **Ref.** | **31** | **Ref.** |
| Serum Antibodies | | | | | | | | | | | | | | | |
| IgG (mg/dL) | **6.3** | | 6.65-13 | **5.85** | 6.65-13 | **3.58** | 6.65-13 |  |  | 10.68 | 6.65-12.78 | 9.66 | 6.65-12.78 | 10.62 | 6.65-12.78 |
| IgA (mg/dL) | **0.19** | | 0.7-3.44 | **0.23** | 0.7-3.44 | **<0.24** | 0.7-3.44 |  |  | **0.30** | 0.7-3.45 | **0.44** | 0.7-3.45 | **0.35** | 0.7-3.45 |
| IgM (mg/dL) | **0.4** | | 0.5-2.09 | 0.54 | 0.5-2.09 | **<0.16** | 0.5-2.09 |  |  | 0.77 | 0.5-2.09 | 0.82 | 0.5-2.09 | 0.64 | 0.5-2.09 |
| Lymphocytes | | | | | | | | | | | | | | | |
| Total lymphocytes  (/μL) | 1300 | |  | 1900 |  | 1900 |  | 1881 |  | 1755 | 1300-4400 | 1966 | 1300-4400 | 2241 | 1300-4400 |
| T cells | | | | | | | | | | | | | | | |
| CD3+ (%) | 84 | |  | 84 | 56-84 | 82 | 56-84 | 77 | 64-85 | 76.6 | 64-85 | 1549.21 | 810-1850 | 76.9 | 64-85 |
| CD3+ (/μL) | 1596 | |  | 1596 | 1000-2200 | 1558 | 1000-2200 | 1448 | 807-1844 | 1359.65 | 810-1850 | 78.8 | 64-85 | 1723.33 | 810-1850 |
| CD4+ (%) |  | |  | 51 | 31-52 | 43 | 31-52 | 35 | 34-62 | 38 | 34-62 | 38.4 | 34-62 | 36.2 | 34-62 |
| CD4+ (/μL) |  | |  | 969 | 530-1300 | 817 | 580-1300 | 658 | 460-1232 | 674.5 | 460-1230 | 754.94 | 460-1230 | 811.24 | 460-1230 |
| CD8+ (%) |  | |  | 28 | 18-35 | 31 | 18-35 | 31 | 14-42 | 29.7 | 14-42 | 31.1 | 14-42 | 30.6 | 14-42 |
| CD8+ (/μL) |  | |  | 532 | 330-920 | 589 | 330-920 | 583 | 187-844 | 527.17 | 190-850 | 611.43 | 190-850 | 685.75 | 190-850 |
| CD45RO+CD4+ (%) |  | |  | 53 |  | 40 |  | 61 |  |  |  | **43.6** | 50-70 |  |  |
| CD45RA+/CD4+ (%) |  | |  | **47** | 58-70 | 60 | 58-70 | 39 | 20-86 |  |  | **56.4** | 30-50 |  |  |
| CD31+/CD4+ (%) |  | |  | 35 |  | 40 |  | 35 |  |  |  | 40.3 |  |  |  |
| CD31+CD45RA+/  CD4+ (%) |  | |  | **27** | 43-55 | **37** | 43-55 | **27** | 30-48 |  |  | 34.3 | 30-48 |  |  |
| CD45RA+CCR7+/  CD8+ (%) |  | |  |  |  | **71** | 51.75-67.58 | **59** | 37-50 |  |  | **56** | 37-50 |  |  |
| CD45RA-CCR7+/  CD8+ (%) |  | |  |  |  | **1** | 2.59-4.19 | **4** | 6-16 |  |  | **1.3** | 6-16 |  |  |
| CD45RA-CCR7-/  CD8+ (%) |  | |  |  |  | 12 | 11.06-19.72 | **23** | 25-37 |  |  | **10.3** | 25-37 |  |  |
| CD45RA+CCR7-/  CD8+ (%) |  | |  |  |  | **15** | 16.21-28.46 | 14 | 8-20 |  |  | **32.4** | 8-20 |  |  |
| CD45RO+/CD8+ (%) |  | |  | 9 |  |  |  |  |  |  |  |  |  |  |  |
| CD45RA+/CD8+ (%) |  | |  | 94 |  |  |  |  |  |  |  |  |  |  |  |
| CD31+/CD8+ (%) |  | |  | 92 |  |  |  |  |  |  |  |  |  |  |  |
| CD31+CD45RA+/  CD8+ (%) |  | |  | 88 |  |  |  |  |  |  |  |  |  |  |  |
| CXCR3+CCR5-/CD4+RA- (%) |  | |  |  |  |  |  |  |  |  |  | 51.3 | 43.4-61.6 |  |  |
| CCR4+CCR6-/  CD4+RA- (%) |  | |  |  |  |  |  |  |  |  |  | **2.4** | 3.8-11.4 |  |  |
| CCR4+CCR6+/  CD4+RA- (%) |  | |  |  |  |  |  |  |  |  |  | **0.8** | 4.9-13 |  |  |
| CXCR5+/  CD4+RA- (%) |  | |  |  |  |  |  |  |  |  |  | **38.1** | 17-29 |  |  |
| CD25+CD127lo/  CD4+ (%) |  | |  |  |  |  |  |  |  |  |  | **3.7** | 5.1-10.3 |  |  |
| B cells | | | | | | | | | | | | | | | |
| CD19+ (%) | 14 | 6-23 | | 12 | 6-23 | 13 | 6 | 10 | 6.6-10.8 | **11.8** | 7.2-11.2 | 11.2 | 7.2-11.2 | **12.2** | 7.2-11.2 |
| CD19+ (/μL) | 182 | 110-570 | | 228 | 110-570 | 247 | 276 | 188 | 133-255 | 209.45 | 92-420 | 220.19 | 92-420 | 273.4 | 92-420 |
| CD21hi+CD24+/  CD19+ (%) |  |  | |  |  |  |  |  |  | 73.8 |  | 79.8 |  |  |  |
| CD21-CD38-/  CD19+ |  |  | |  |  |  |  |  |  | **0.05** | 1.8-4.7 | **1.3** | 1.8-4.7 |  |  |
| CD24hi+CD38hi+/  CD19+ (%) |  |  | |  |  |  |  |  |  | **5** | 1-3.6 | 3.3 | 1-3.6 |  |  |
| CD24hiCD27-CD38hi+IgD+/  CD19+ (%) |  |  | |  |  |  |  |  | 4.3 |  | 2.7 |  |  |  |  |
| CD24-CD38hi+/CD19+ (%) |  |  | |  |  |  |  |  |  | **0.05** | 0.6-1.6 | **0.4** | 0.6-1.6 |  |  |
| CD27-IgD+/CD19+ (%) |  |  | |  |  |  |  | **87** | 65.6-79.6 | **81.7** | 58-72.1 | **83.3** | 58-72.1 |  |  |
| CD27+/CD19+ (%) | 11 | >10 | | **8** | >10 | **4** | 8 | 11 |  | 16.7 |  | **15.8** |  |  |  |
| CD27+/CD19+ (/μL) | 20 |  | | 18 |  | 10 | 18 | 21 |  |  |  |  |  |  |  |
| CD27+IgD+/CD19+ (%) |  |  | |  |  |  |  | 8 | 7.4-13.9 | 13.8 | 13.4-21.4 | **11.6** | 13.4-21.4 |  |  |
| CD27+IgD-/CD19+ (%) |  |  | |  |  |  |  | **2** | 7.2-12.7 | **3** | 9.2-18.9 | **2.9** | 9.2-18.9 |  |  |
| NK cells | | | | | | | | | | | | | | | |
| CD16+CD56+ (%) |  |  | | 4 | 3-22 | 4 | 4 | 11 | 5-20 | 11.4 | 5-20 | 9.9 | 5-20 | 10.7 | 5-20 |
| CD16+CD56+ (%) |  |  | | 76 | 70-480 | 76 | 76 | 207 | 89-362 | 202.35 | 89-362 | 194.63 | 89-362 | 238.79 | 89-362 |
| Impression: Hypogammaglobulinemia. Low naïve T cells, particularly recent thymic emigrants. Elevated naïve B cells, low memory B cells, and very low class-switched memory B cells | | | | | | | | | | | | | | | |

| Patient 9 | | | | | | | |  | | Patient 10 | | | | |
| --- | --- | --- | --- | --- | --- | --- | --- | --- | --- | --- | --- | --- | --- | --- |
| Age (months) | **4** | **Ref.** | **24** | **Ref.** | **36** | **Ref.** |  | | **Age (years)** | | **5.5** | | **Ref.** | |
| Serum Antibodies | | | | | | | |  | | **Serum Antibodies** | | | | |
| IgG (mg/dL) | **Very low** |  | **286** | 385-1411 | **341** | 385-1411 |  | | IgG (mg/dL) | | **354.8** | | 463-1236 | |
| IgA (mg/dL) | **Very low** |  | **<26** |  | **<26** |  |  | | IgA (md/dL) | | **21.2** | | 43-196 | |
| IgM (mg/dL) | **Very low** |  | **<18** |  | **<18** |  |  | | IgM (mg/dL) | | **18** | | 25-154 | |
| IgE (IU/mL) |  |  |  |  | 2 |  |  | | IgE (IU/mL) | | **316.5** | | 1.03-161 | |
| T cells | | | | | | | |  | | **Leukocytes** | | | | |
| CD3+ (%) | Normal |  | 27.2 | 56-75 |  |  |  | | Leukocytes (/μL) | | 10300 | |  | |
| CD4+ (%) |  |  | 30.5 | 32-51 |  |  |  | | **Impression: Hypogammaglobulinemia. No detailed immunophenotyping available** | | | | | |
| CD8+ (%) |  |  | <5.3 | 14-30 |  |  |  | |  |  |  |  |  |  |
| CD4:CD8 | **High** |  |  |  |  |  |  | |  | | | | | |
| CD45RA+CD3+ (%) |  |  | 74.87 |  |  |  |  | |  | |  | |  | |
| CD45RO+CD3+ (%) |  |  | 20.6 | 20-46 |  |  |  | |  | |  | |  | |
| CD45RA+CD3+CD4+ (%) |  |  | 74.7 | 46-77 |  |  |  | |  | |  | |  | |
| CD45RO+CD3+CD4+ (%) |  |  | 23.6 | 13-30 |  |  |  | |  | |  | |  | |
| CD45RA+CD3+CD8+ (%) |  |  | 81.2 | 63-92 |  |  |  | |  | |  | |  | |
| CD45RO+CD3+CD8+ (%) |  |  | **26.6** | 4-21 |  |  |  | |  | | | | | |
| Th17 (%) | **Low** |  |  |  | **0.85** | 3.6 |  | |  | |  | |  | |
| B cells | | | | | | | |  | |  | |  | |  |
| CD19+ | Normal |  |  |  |  |  |  | |  | |  | |  | |
| CD19+27-IgD+ (%) |  |  | 40.93 | 42.19 |  |  |  | |  | |  | |  | |
| CD19+27+IgD+ (%) |  |  | 57.87 | 49.89 |  |  |  | |  | |  | |  | |
| CD19+27+IgD- (%) |  |  | **1.2** | 7.78 |  |  |  | |  | |  | |  | |
| NK cells | | | | | | |  | |  | |  | |  | |
| NK cells (%) | **Low** |  |  |  |  |  |  | |  | |  | |  | |
| Other | | | | | | | |  | |  | |  | |  |
| Lymphocyte proliferation to PHA | Normal |  |  |  |  |  |  | |  | |  | |  | |
| Impression: Hypogammaglobulinemia. Elevated CD4+ T cells with a high CD4:CD8 ratio, reduced memory CD8+ T cells, and reduced Th17. Low class-switched memory B cells | | | | | | | |  | |  | |  | |  |

**Supplemental Table 3: Immunophenotyping data of P8-10.** Ref. - Reference value.
