# Supplementary material for: IRF4 p.T95R combined immunodeficiency: Clinical features and transplant outcomes
Source: J Hum Immun. 2026 Oct 9;2(6):e20260069. doi: 10.70962/jhi.20260069 (PMC13651748; doi:10.70962/jhi.20260069)
Supplement: Supplementary file 4 [file jhi_20260069_tables4.docx]

| **Variant** | **Inheritance**  **(Mechanism)** | **Phenotype** | **Databases** | | ***In Silico* Models** | | | | |
| --- | --- | --- | --- | --- | --- | --- | --- | --- | --- |
|  |  |  | **ClinVar** | **GnomAD** | **CADD** | **Alpha missense** | **REVEL** | **Splice AI** |  |
| c.284C>G, p.T95R^1^ | AD  (MM) | Fatal CID | Pathogenic | Not found | Deleterious  (24.9) | Deleterious  (0.999) | Deleterious  (0.8) | Benign  (0) |  |
| c.1075 T>C, p.F359L^2^ | AD  (MM) | CID | Pathogenic | Not found | Deleterious (21.5) | Deleterious  (0.856) | Uncertain  (0.52) | Benign  (0) |  |
| c.292 C>T, p.R98W^3^ | AD  (LOF) | WD | Pathogenic | Not found | Deleterious  (21.6) | Deleterious  (1) | Deleterious  (0.8) | Benign  (0) |  |
| c.73C>A, p.R25S^4^ | AD  (LOF) | WD | Not found | Not found | Deleterious (25.8) | Deleterious  (1) | Deleterious  (0.93) | Benign  (0) |  |
| c.1213-2A>G, p.V405GfsTer127^5^ | AR UPD  (LOF) | Fatal CID +  Syndromic features* | Not found | Not found | Deleterious  (33) | N/A | N/A | Splice-altering (0.36) |  |

**Supplemental Table 4: Phenotypic and *in silico* characteristics of known damaging IRF4 variants.** CADD - Combined Annotation-Dependent Depletion; REVEL - Rare Exome Variant Ensemble Learner; AD – autosomal dominant; MM – multimorphic; LOF – loss of function; AR – autosomal recessive, UPD – uniparental isodisomy; CID – combined immunodeficiency, WD – Whipple’s disease. ^1^(14); ^2^(34); ^3^(12); ^4^(38); ^5^(1); *Intrauterine growth retardation, failure to thrive, hypoglycemia, tachycardia, fevers, generalized adenopathy, and dermatitis.
